# Gut consciousness: a perspective on visceral phenomenal awareness

**DOI:** 10.1093/nc/niag070

**Published:** 2026-10-26

**Authors:** Gregor Hasler

**Affiliations:** Molecular Psychiatry Lab, Faculty of Science and Medicine, University of Freiburg, FNPG, Chemin du Cardinal-Journet 3, 1752 Villars-sur-Glâne, Switzerland; University Psychiatry Research Unit, Freiburg Mental Health Network, Chemin du Cardinal-Journet 3, Villars-sur-Glâne 1752, Switzerland; Lake Lucerne Institute, Rubistrasse 9, 6354 Vitznau, Switzerland

**Keywords:** enteric nervous system, interoception, predictive processing, embodied cognition, microbiota–gut–brain axis, pre-reflexive self

## Abstract

The proposition that the enteric nervous system sustains a form of consciousness is seldom entertained in mainstream neuroscience, yet it follows from a disciplined reading of contemporary theories of mind. We argue that the 400–600 million neurons embedded in the mammalian gastrointestinal wall, taken together with the vast interoceptive surface they supervise, the enteroendocrine–immune interface, and the microbial community they co-regulate, constitute a substrate that satisfies, on several of the leading theories of consciousness, the criteria those theories use to attribute experience elsewhere. We synthesize evidence on enteric autonomy, chemo- and mechanosensory resolution, vagal and humoral signalling, self/non–self-discrimination at the epithelial–immune boundary, comparative data from invertebrates whose nervous systems are dominated by gut-like plexuses, and convergent clinical signatures (irritable bowel syndrome, food intolerances, psychedelic nausea, glucagon-like peptide-1 signalling) that are difficult to accommodate under a purely sensorimotor account. Throughout we distinguish two claims that are easily conflated: that the gut makes a large and underappreciated contribution to the single consciousness of the whole animal, and that enteric processing constitutes a second, much simpler locus of experience of its own. Most of the available evidence bears on the first, and we set out five lines of investigation that would discriminate between them. We conclude that gut consciousness is a testable hypothesis, likely corresponding to an ‘animal’ mode of consciousness dominated by interoceptive feel, drive and affect, and we propose empirical directions for its further investigation.

## Introduction

Consciousness research has, for most of its modern history, been an investigation of the head. Cortical correlates, thalamocortical loops, prefrontal ignition, the claustrum and the posterior ‘hot zone’ dominate the empirical literature (Dehaene and Changeux 2011, Koch et al. 2016). The body appears, if at all, only as content to be represented: a map, an afferent stream, a set of error signals that a central inferential machine must reconcile. Even the phenomenological tradition, which placed the lived body at the heart of its enterprise, has had almost nothing to say about the viscera. In Merleau-Ponty’s *Phenomenology of Perception* (Merleau-Ponty 1945), arguably the twentieth-century canon of embodied experience, the gut is not mentioned once.

This silence should not be read as an oversight, and we do not present it as one. Phenomenology describes, on principle, what is manifest in subjective experience, and the gut does not manifest itself as a subject there. Its near-absence from the phenomenological canon is what the method predicts rather than something the method missed. But this is precisely what makes the present question hard, and the difficulty is better stated at the outset than at the end: if enteric processing sustains any experience of its own, that experience is by hypothesis not given to the person whose gut it is, and first-person description cannot reach it. What the silence establishes is therefore modest—that the question of visceral subjectivity has been left unasked rather than answered—and the burden of asking it falls to methods other than description from the inside.

Two developments of the past three decades invite a broader ontology. First, the leading theories of consciousness have become increasingly substrate-neutral. Integrated information theory (IIT) (Tononi 2004, Koch et al. 2016) predicts phenomenal experience wherever causal integration reaches a sufficient threshold, irrespective of anatomical location. Global workspace theory (GWT) (Baars 1988, Mashour et al. 2020) localizes consciousness not in a region but in the functional availability of information for broadcast and behavioural control. Predictive processing and the free-energy principle (Friston 2010, Clark 2013) recast consciousness as the active inference of an organism’s hidden causes, including its own interior. Embodied and enactive approaches (Varela et al. 1991, Thompson 2007) return body and world to the centre of the mental. Together these frameworks strip the cortex of its metaphysical privilege.

Second, neurogastroenterology has revealed that the enteric nervous system (ENS), once caricatured as a peripheral reflex circuit, is a computational organ of extraordinary sophistication. Approximately 400–600 million neurons (Gershon 1998, Furness 2012), organized into myenteric and submucosal plexuses, innervate a digestive surface that reaches, with its villi, some 30 m^2^, comparable in area to a singles tennis court (Helander and Fändriks 2014). The ENS contains every major neurotransmitter class found in the brain, synthesizes roughly 90–95% of the body’s serotonin (Gershon and Tack 2007), and can execute complex motor programmes entirely in the absence of central input (Furness 2012, Spencer and Hu 2020). Michael Gershon’s designation of the ENS as a ‘second brain’ (Gershon 1998) has, if anything, understated the matter: few other peripheral systems come close in neuronal count, behavioural autonomy, or information-theoretic complexity.

The conjunction of these two developments—substrate-neutral theories of consciousness on one side, a computationally competent and partially autonomous neuronal organ on the other—raises a question that has been curiously neglected: does the gut, in some minimal but non-trivial sense, have a phenomenal life of its own? We do not adjudicate between the frameworks. We make the more modest and more robust claim that the plausibility of gut consciousness is strengthened precisely because it survives scrutiny from several of them at once. That the gut has been culturally subordinated, associated with waste and disgust, and excluded from the ennobled precincts of mind bears on none of this.

### What is claimed and what is not: two theses about the gut

Because the argument turns on distinctions that are easily blurred, we state at the outset what is being claimed. By consciousness we mean phenomenal consciousness in Nagel’s sense: there is something it is like for the system to be in the states it is in (Nagel 1974). It is worth spelling out what that formula entails, since much of what follows depends on it. To say that there is something it is like for a system is to say that the system has subjective experience, and subjective experience has at least two basic and interdependent features. The first is intentionality: an experience is of or about something, so that the state is an awareness of a content—of distension, of sourness, of a breach in the wall. The second is minimal self-awareness: in undergoing the state, the subject is aware of being aware. In the Husserlian tradition this second feature is understood pre-reflectively, pre-conceptually and pre-linguistically. It is not introspection, not a thought about oneself, and not a self-concept, but the bare first-personal givenness in virtue of which an experience is had by someone at all (Zahavi 2005, Gallagher and Zahavi 2012). The two features are interdependent: awareness of being aware requires being aware of something, and an awareness of something that was had by no one would not be an experience. Nothing counts as conscious that lacks either.

Following Block (1995), we separate this from access consciousness: the availability of a content for reasoning, verbal report and the deliberate control of action. Nagel’s criterion is not a theory of consciousness that competes with the frameworks discussed in Section 4. It specifies the explanandum that those frameworks attempt to explain, and we treat it accordingly throughout.

What we attribute to the ENS, on the stronger reading of our thesis, is phenomenal consciousness of this minimal kind: valenced interoceptive states—comfort and discomfort, urgency and quiescence, tolerance and rejection—directed upon a luminal and mural content and given pre-reflectively to whatever undergoes them. We do not attribute access consciousness, reportability, reflective self-consciousness, a self-concept, a narrative self, propositional thought, or any capacity to represent objects at a distance. Any of these would be extravagant, and none is required by what follows. What remains is nonetheless a real requirement and not a formality: an organ that merely regulated, however elaborately, without any of its states being *for* it would not qualify, and we do not claim that the evidence below establishes that the enteric case is not of that kind. What it establishes is that the ENS possesses, to an unusual degree, the functional properties that on every current theory accompany minimal awareness where we independently accept it.

With the explanandum fixed, two claims must be separated that are easily run together. The first we call the **contributory thesis**: enteric processing makes a large, structured and underappreciated contribution to the consciousness of the whole animal, so that a substantial part of what it is like to be a human being is constituted by states that originate in, and are pre-processed by, the gut. The second we call the **partial-subject thesis**: enteric processing constitutes a locus of experience of its own, so that in addition to the familiar whole-organism subject there is, within the same body, a second and far simpler subject whose experiences are not the person’s experiences. The two theses are logically independent. The contributory thesis can be true while the partial-subject thesis is false, and that is the default reading of contemporary interoceptive and embodied accounts of experience (Damasio 1999, Craig 2002, Damasio and Carvalho 2013, Seth 2013), which enrich the single subject rather than multiply subjects. The reverse combination is coherent but less plausible.

Most of the evidence assembled below bears directly on the contributory thesis and only indirectly on the partial-subject thesis. The scale and autonomy of the enteric plexuses, the resolution of enteric chemosensation, the speed of enteroendocrine–vagal transduction and the clinical phenomena of Section 8 all show that the gut computes a great deal, and hence that whatever consciousness exists in the organism is more visceral than the cortex-centred literature assumes; none of it is by itself evidence of a second subject. Our position is accordingly graded. The contributory thesis we regard as well supported and as sufficient on its own to justify a reorientation of research attention toward the gut. The partial-subject thesis we regard as a live and testable candidate rather than an established conclusion, and Section 9 specifies what would have to be observed for it to be supported or defeated.

The partial-subject thesis conflicts with a widely held assumption that consciousness is unified: that at any moment an animal has a single phenomenal field, and that its many parallel processes furnish contents of that field rather than fields of their own (Bayne 2010). Many readers will find the alternative not merely improbable but disturbing, and we do not think that reaction unreasonable. It is, however, an assumption rather than a finding, and one questioned from within consciousness science (Schechter 2018, Godfrey-Smith 2021). We take it up as the fourth objection in Section 10.

Sections 2 and 3 review the neurobiological facts that make the ENS a candidate at all and that distinguish it from the organ systems for which no such claim is made. Section 4 evaluates the hypothesis against four theoretical frameworks—IIT, GWT, predictive processing, and the marker-based treatment of self-modelling and behavioural flexibility—treating Nagel’s criterion, for the reasons given above, as a statement of the explanandum rather than as a fifth framework. Sections 5 to 8 position the gut within embodied cognition, immunological self/non–self-discrimination, comparative biology, and the clinic. Section 9 sets out how the two theses could be told apart empirically, which we take to be the central methodological question raised by the proposal, and Section 10 addresses objections. Our claim is deliberate and restricted: we do not argue that the gut reasons in language or entertains concepts of a reflexive self. We argue that the ENS contributes centrally to an ‘animal’ consciousness—a stream of drive, affect and interoceptive feel—and that it is a serious candidate for sustaining such a mode locally.

## The ENS: autonomy, scale, and the contrast with other organ systems

Three features of the ENS make it unlike any other peripheral innervation. First, numerical scale: estimates place the neuron count between 200 and 600 million, exceeding that of the spinal cord and roughly matching a cat’s cortex (Gershon 1998, Furness 2012). Second, cytological and neurochemical diversity: at least 17 distinct enteric neuron classes have been described, deploying acetylcholine, serotonin, nitric oxide, vasoactive intestinal peptide, substance P, GABA, dopamine and many others (Furness 2000, Spencer and Hu 2020). Third, and most consequential, functional autonomy: isolated segments of intestine, entirely disconnected from brain and spinal cord, generate coordinated peristaltic, segmenting and migrating-motor-complex patterns (Furness 2012, Spencer and Hu 2020). Sympathetic and parasympathetic fibres modulate the ENS but do not instruct it in any detailed sense.

These comparisons matter because the claim under examination is not that the gut is complicated. Kidney, liver and pancreas are complicated. The claim is that on the dimensions that consciousness theories treat as relevant, the enteric plexuses belong closer to the brain than to the other viscera. The intrinsic cardiac nervous system, the only serious competitor, comprises on the order of tens of thousands of neurons in ganglionated plexuses with genuine local reflex capability (Armour 2008)—real autonomy, but three to four orders of magnitude below the enteric case and with a far narrower behavioural repertoire. No other organ system contains a neuronal population of this size, a comparable diversity of neuron classes, or a comparable capacity to generate patterned, long-duration output after extrinsic denervation (Furness et al. 2014).

The direction of traffic is often underappreciated. The vagus nerve, historically viewed as a top-down conduit of central control, is now understood to be overwhelmingly afferent: roughly 80% of its fibres carry information from gut to brain (Godfrey-Smith 2016, Breit et al. 2018). The brain is, on this view, a largely reactive listener to an organ that makes most of its own decisions; and when the central nervous system is acutely lesioned, as in high spinal transection, gastrointestinal motility reorganizes but does not stop (Spencer and Hu 2020). No comparable independence is exhibited by heart, kidney or lung.

Enteric neurons, like central neurons, are predominantly post-mitotic (Rao and Gershon 2016). An individual neuron does not divide once established; it accumulates experience across the lifespan. Every human gut is therefore, at the neuronal level, a unique historical record of what has been eaten, what has been survived, and with which microbes a truce has been struck. The ENS is thus a learning system whose plasticity is constrained as the brain’s is, which separates it from the organ systems to which no consciousness claim is made, whose function rests largely on mitotically renewing epithelial, immune and endocrine populations. Adult enteric neurogenesis remains debated, so this should be read as a claim about the predominant mode of enteric plasticity rather than an absolute (Rao and Gershon 2016)—and in any case as a claim about plasticity and individuation, not about phenomenality. A learning system need not be an experiencing one, and we do not argue from the one to the other.

A further remarkable property is the system’s spatial extent. Coordinated contractions propagate over the several metres of the human gut, and the enteric plexuses must integrate afferent information from the entire length simultaneously to produce behaviour such as the migrating motor complex, which sweeps undigested residue in a coordinated wave lasting up to 90 minutes (Spencer and Hu 2020). Long-range temporal coordination of this kind is a hallmark not of reflex arcs but of genuinely global computation.

A final point concerns the organ’s accessibility. The brain can be operated upon under local anaesthesia; the patient reports, converses, identifies faces. The bowel cannot be instrumented in an awake, externalized way, and from outside it is reached only through the vagus, the spinal afferents and the bloodstream. This is a liability of the hypothesis rather than a point in its favour, and it is why the discriminating tests of Section 9 rely on markers rather than on report.

## Interoception: the body’s largest sensory surface

Interoception, the sensing of internal bodily states, is now widely regarded as foundational to conscious experience. Damasio’s somatic-marker hypothesis (Damasio 1999, Damasio and Carvalho 2013), Craig’s anatomy of the ‘feeling self’ (Craig 2002) and Seth’s interoceptive-inference extension of predictive processing (Seth 2013) converge on the claim that what it feels like to be an organism is largely constituted by what that organism represents of its own interior. If interoception is that central, the gut deserves first-chair attention.

By any quantitative measure the gastrointestinal tract is the body’s interoceptive powerhouse. The mucosal surface carries a greater density and diversity of sensors than any other internal organ: mechanoreceptors tracking wall tension and stretch, chemoreceptors for glucose, amino acids, fatty acids, bile acids and bacterial metabolites, thermoreceptors, osmoreceptors and nociceptors (Mayer 2011, Mayer et al. 2023). Enteroendocrine cells, historically called simply ‘hormone-secreting cells’, have turned out to be *bona fide* neuroepithelial elements, forming synaptic contacts with vagal afferents and transducing luminal chemistry into rapid neural signals on the millisecond timescale (Bohórquez et al. 2015, Kaelberer et al. 2018). Kaelberer and colleagues (Kaelberer et al. 2018) showed that enteroendocrine cells signal to the brain through a glutamatergic synapse that rivals, in speed and precision, sensory synapses of the retina.

This sensory apparatus is not, contrary to older textbook accounts, a one-way pipeline to the brain. Most afferent traffic terminates locally within the myenteric and submucosal plexuses and never reaches the CNS (Furness 2012, Spencer and Hu 2020). The ENS has its own interoceptive world, which it acts upon through a tightly coupled sensorimotor architecture: every peristaltic contraction is itself a probing of the luminal state, whose consequences are re-sensed, reintegrated, and reacted to. In enactivist terms (Varela et al. 1991), the gut does not merely receive sensation; it dynamically interacts with its sensed milieu through its own movements. This closed-loop coupling is one of the strongest marks that enactive and embodied theories use to identify a cognitive agent.

In one respect the gut surpasses the cephalic brain: it senses chemistry directly to a considerably greater extent. Cortical neurons read the world through layered transductions—photoreceptors, cochlear hair cells, olfactory epithelium—all of which dwell behind the blood–brain barrier. The gut, by contrast, is in direct chemical contact with everything the organism ingests and with much of what its microbiota produces, and the bandwidth and specificity of this chemical channel are, in relevant respects, richer than any sensory modality available to the head. The chemical nature of the ingested environment, and its transformation in the gut, is accordingly a likely content of gut consciousness.

## Gut consciousness across theories of consciousness

Sections 2 and 3 described what the gut does—its scale, its autonomy, its sensory resolution, its closed-loop coupling to a chemically rich milieu—without reference to any theory of consciousness. We now ask how those facts look through four of the frameworks that structure the field, used as independent tests of a candidacy claim rather than as authorities whose verdicts settle the matter; nothing below depends on any of them being true. Our strategy is not to prove that the gut is conscious, which lies beyond the methodological reach of any current theory, but to show that on the very criteria these theories use to attribute consciousness elsewhere, the gut is at least a candidate—on some a strong one, on one a weak one. Methodologically we follow the precautionary logic of Birch (2024): what matters is whether a system qualifies as a credible candidate on grounds that several frameworks, applied independently, would each recognize. Table 1 summarizes the comparison. Throughout, the distinction of Section 1.1 should be kept in view: satisfying a framework’s criteria shows at most that enteric processing has the properties that framework takes to matter, and further argument of the kind set out in Section 9 is needed before this counts in favour of a second locus of experience rather than a richer single one.

**Table 1:** Major theoretical frameworks of consciousness applied to the enteric nervous system

Framework	Core requirement for consciousness	Application to the enteric nervous system	Fit with current evidence	Open challenges
Integrated Information Theory (IIT)	A local maximum of integrated information (Φ) constituting an irreducible complex.	The ENS forms a densely interconnected ganglionic network with recurrent connectivity and partial autonomy from the central nervous system. The question is whether enteric processing reaches a Φ maximum that is not subsumed by the larger CNS-plus-ENS system.	ENS autonomy (peristaltic reflex without CNS), recurrent local processing and distinct rhythmic generators are consistent with a candidate local complex, but quantitative Φ estimates are not yet feasible *in vivo*.	Practical measurement of Φ in biological systems is unresolved. Because of the exclusion postulate, the decisive question is not the absolute value of enteric integration but whether the ENS is a local maximum or is subsumed within the joint ENS–CNS complex (Sections 4.1 and 9).
Global Workspace Theory (GWT)	Selective broadcasting of information across a workspace enabling flexible report and behavioural control.	The ENS lacks a cortical-style workspace. Vagal afferents and enteroendocrine signalling mediate selective transmission, while motility, secretion and immune output are coordinated across the gut.	Limited. On a strict reading—competition, selection, ignition, broadcast—the ENS has not been shown to instantiate a workspace at all. This is the weakest fit among the frameworks considered (Section 4.2).	GWT in non-cortical and distributed peripheral systems is underdeveloped. A workspace-equivalent concept for autonomous nervous tissue is needed, together with recordings of candidate ignition events.
Predictive processing and active inference	Hierarchical generative models that minimize prediction error through perception and action.	The ENS continuously predicts and adjusts to luminal contents, microbial activity and mechanical state. Functional gastrointestinal disorders are interpretable as miscalibrated enteric generative models.	Strong conceptual fit, but the framework is permissive. Emerging support from interoceptive predictive coding and from clinical models of disordered gut sensation.	Distinguishing reflex chains from genuine inference: precision-weighting, anticipation and model revision must be demonstrated rather than assumed (Section 4.3).
Embodied and enactive cognition	Cognition emerges from sensorimotor coupling and the autonomous self-maintenance of a living system.	The ENS is a paradigm of autonomous, self-organising sensorimotor activity. The holobiont (gut plus microbiota) extends the boundary of the cognising system.	Strong. ENS autonomy and microbiota–gut interactions satisfy operational closure and structural coupling criteria.	These are theories of the vehicle of one subject’s cognition; they are compatible with the contributory thesis and silent on the partial-subject thesis (Section 5).
Nagel’s ‘what it is like’ criterion (not a theory of consciousness but a specification of the explanandum; listed for completeness and discussed as such in Sections 1.1 and 4.4)	There must be something it is like to be the system: intentional directedness upon a content together with minimal, pre-reflective self-awareness.	Cannot be settled empirically alone. Precautionary inference from convergent markers supports the credibility of asking the question.	Indirect but cumulative. The gut meets several behavioural and physiological correlates that justify treating it as a credible candidate.	Inherent epistemic limits of subjective ascription to non-reporting systems. The boundary between functional analogy and phenomenal reality.
Birch’s theory-light marker framework	Convergent behavioural and physiological markers of sentience without commitment to a single theory of consciousness.	The ENS displays multiple markers: nociceptive sensitisation, conditioned learning, opioid-modulated analgesia, integration of chemosensory and mechanosensory input into context-sensitive output.	Several markers documented. The cluster meets the precautionary threshold for a credible candidate.	The marker list is still being refined. Weighting and minimum-cluster criteria remain contested. Comparative work across invertebrates and the ENS is sparse.

Each row applies one framework to enteric processing on its own terms, lists the empirical fit, and notes outstanding theoretical or methodological challenges. The table is intended to be read alongside the precautionary, theory-light marker logic of Birch (2024). Fit assessments are interpretive and reflect the convergent rather than decisive nature of the evidence; they concern candidacy on each framework’s own criteria, and none of them, on its own, distinguishes the contributory from the partial-subject thesis (Sections 1.1 and 9).

### Integrated information theory

IIT (Tononi 2004, Tononi et al. 2016) proposes that consciousness is identical to integrated information, formalized as the non-decomposable causal structure Φ. A system is conscious to the extent that its causal powers upon itself cannot be reduced to the independent contributions of its parts. Three features of the ENS make it a promising candidate. First, its neuron count and synaptic density permit very large state spaces. Second, the plexuses form recurrent, mutually constraining networks whose behaviour demonstrably cannot be explained by summation of independent units, since the coordination of long-range motor patterns requires integration (Spencer and Hu 2020). Third, the ENS is physically closed enough to admit a principled ‘complex’ analysis: its main causal frontiers—vagal afferent and efferent, spinal sympathetic, blood-borne—are identifiable and sparser than internal ENS connectivity. Whether Φ has actually been computed for enteric networks is an open empirical question, but IIT’s mathematics does not reject such a measurement in principle.

IIT’s exclusion postulate, however, constrains the reading of our thesis more sharply than any other framework does. On IIT, experience belongs to a maximum of intrinsic irreducibility, and overlapping candidate systems cannot both be conscious: if the ENS and the CNS together form a single complex, enteric elements contribute to the person’s experience and constitute none of their own (Oizumi et al. 2014). The substantive question is therefore not whether enteric integration is large in absolute terms but whether the ENS is its own maximum or is subsumed within the joint ENS–CNS system. That turns on the relative density and effectiveness of internal as against crossing connections, and here the anatomy is at least suggestive: on the order of 100 000 vagal fibres, together with a comparably sparse spinal projection, connect several hundred million enteric neurons to the brain—a bottleneck far narrower than any that separates two cortical areas. Whether such a bottleneck suffices to sever a complex is not currently decidable, but it is a well-posed question that the formalism itself can be made to answer, and it is the same question as the partial-subject question (Section 9).

### Global workspace theory

GWT (Baars 1988, Mashour et al. 2020) locates conscious contents in a limited-capacity workspace whose broadcasts coordinate otherwise specialized processors. Digestion looks at first like a textbook case. Over a post-prandial interval of several hours, information about luminal nutrients, wall tension, pH, osmolarity, microbial metabolites and immune signals must be integrated to coordinate gastric emptying, pancreatic exocrine release, biliary flow, segmental motility, absorption and elimination. No single ganglion manages this; the plexuses together must maintain something like a shared, contentful space whose updates modulate multiple effector systems (Chambers et al. 2013, Spencer and Hu 2020), and maintenance of the microbiome across weeks and months represents a still longer-horizon working memory (Cryan et al. 2019).

What this shows, however, is integration, and integration is not yet a workspace. A global workspace in the sense GWT intends is a specific architecture: a limited-capacity resource for which candidate contents compete, a selection mechanism, a non-linear ignition in which the winning content is amplified and sustained, and a broadcast of that content to consumer processes that could not otherwise access it (Mashour et al. 2020). What we have described in the gut is a clearing house—a system in which many streams of information converge and are reconciled into coherent output. A clearing house is compatible with GWT but does not instantiate it, and the ENS may well accomplish its work by a quite different architecture in which distributed local reconciliation never requires a global bottleneck at all. The fit with GWT is accordingly the weakest of the frameworks considered here.

There is nonetheless a testable enteric analogue of ignition worth naming, and we offer it as a concrete proposal rather than as a claim. Certain enteric events are all-or-none, globally recruiting and suppressive of everything else: the retrograde giant contraction that precedes emesis abolishes ongoing motor programmes along a large fraction of the small intestine and reorganizes secretion, blood flow and sphincter tone within seconds (Lang et al. 1986); giant migrating contractions and the defecatory sequence behave comparably. If GWT is to be applied to the gut seriously, this is where to look, and the relevant experiments ask whether such events exhibit the signatures the theory predicts: competition among candidate triggers, a threshold non-linearity, sustained amplification of the winner, and broadcast to effector systems that are otherwise informationally segregated from one another. Until recordings of that kind exist, the honest verdict is that the ENS integrates but has not been shown to broadcast.

### Predictive processing and active inference

Friston’s free-energy principle (Friston 2010) frames cognition as the minimisation of long-run surprise through active inference. Seth (2013) has extended this to interoception, arguing that emotion is the predictive control of bodily states. On this framework, consciousness is closely related to irreducible uncertainty about hidden causes: the more an organism must infer what it cannot directly control, the more it needs the representational apparatus we call experience. The gut is an ideal candidate. It influences food intake through satiety and nausea signals but does not decide what will enter it. Microbial composition is perturbed unpredictably by diet, stress and pathogen exposure. Absorptive demand is set elsewhere. The gut must therefore continuously generate, update and act on predictions under substantial irreducible uncertainty.

We resist the temptation to quantify this against the cortex, since free energy is not a currency in which organs can be meaningfully totalled. The more useful observation is that predictive processing, unlike IIT, is permissive: reflex arcs and thermostats can be redescribed as minimising prediction error, so a good fit with the framework establishes very little on its own (Clark 2013). What would establish something is evidence for the features that distinguish inference from reflex—precision-weighting, in which the gain on an afferent channel is set by that channel’s estimated reliability; anticipatory responses that precede the stimulus they answer to, as cephalic-phase secretion does (Smeets et al. 2010); and revision of the model rather than repetition of the response when predictions fail. Each of these is measurable in the gut, and each is at present more assumed than demonstrated.

### Minimal phenomenality: what the hypothesis requires of the gut

Nagel (1974) argued that consciousness is whatever is present when there is ‘something it is like’ to be a system. As set out in Section 1.1, this specifies the explanandum rather than a theory of it, and it does not belong on a list with IIT and GWT. What it does is fix what any of those frameworks would have to deliver in the enteric case, and it is worth being explicit about how demanding that is.

The hypothesis requires nothing extravagant from the gut: not concepts of itself, not linguistic articulation, not reflective self-consciousness. It requires the two features set out in Section 1.1—intentional directedness upon a content, and pre-reflective self-awareness—in their most minimal form, which is the form phenomenologists in the Husserlian tradition take to be necessary to any consciousness whatsoever (Zahavi 2005, Gallagher and Zahavi 2012). On that reading a system can be conscious without being able to say so, without any concept of itself, and without ever making itself an object of reflection; what it cannot be is conscious without its states being, in the barest sense, its own. Nothing in Sections 2 and 3 demonstrates that. What those sections demonstrate is that the ENS possesses the functional properties that accompany minimal awareness in every case where we independently accept it, which is what makes the gut a candidate and not a conclusion.

It should also be said plainly where this leaves the hypothesis in relation to existing positions, since it is easy to recruit allies who have not enlisted. Godfrey-Smith allows a minimal case of experience for the box jellyfish, tied to its unusual sensory and motor apparatus rather than to its digestive organisation, and places most cnidarians at or below the threshold (Godfrey-Smith 2016). Goff’s panpsychism does attribute experience to lancelets and echinoderms, but only as a consequence of attributing it to everything, and so privileges the gut no more than it privileges a stone (Goff 2019). There is no existing tradition that supports the claim examined here. That is a reason for caution, and for advancing the claim as a hypothesis with specified tests, which is how we advance it.

### Self-modelling, attention, and behavioural flexibility

Mainstream markers of consciousness include self-modelling (distinguishing organism from environment), attentional flexibility (selective weighting of inputs) and non-reflexive, context-dependent response (Baars 1988, Dehaene et al. 2017). Each is exemplified by the ENS. Self/non–self-discrimination at the epithelial–immune interface is arguably more elaborate than in any other organ (Section 6). The gut selectively weights incoming signals, upregulating lipase secretion for fatty meals, altering motility for high-sugar loads, and gating absorption according to microbial and immune cues (Mayer et al. 2023). Its responses are not reflexes in the spinal sense; they are context-sensitive programmes whose outcome depends on recent meals, circadian state, microbiome composition and stress-axis tone. Attempting to describe gut behaviour in a purely reflex vocabulary fails for the same reason such descriptions have repeatedly failed elsewhere in the study of animal behaviour (Birch 2024): the mapping from stimulus to response is many-to-many and depends on state variables internal to the system.

It is worth being concrete about the behavioural repertoire in question, since the word *behaviour* is doing unfamiliar work here. The output side of the ENS comprises patterned motility (peristalsis, segmentation, the migrating motor complex, giant migrating contractions, receptive accommodation, the retrograde giant contraction and emesis, the defecatory sequence), control of secretion, regulation of mucosal blood flow, gating of epithelial permeability and transport, and modulation of local immune effectors (Furness 2012, Furness et al. 2014, Spencer and Hu 2020). None of this moves the animal through space; all of it is action in the sense that matters to enactive accounts, namely intervention upon a milieu whose subsequent state is then sensed. The temporal grain is unfamiliar as well: enteric programmes unfold over minutes to hours rather than the hundreds of milliseconds of skeletal action, and any comparison of enteric with cortical dynamics that ignores this will mislead in both directions.

Eating is the instructive boundary case. The decision to eat, what to eat and when to stop is made by the whole organism, and if any process deserves to be called an exercise of the person’s agency, this one does. The contribution of the gut is not decision but valuation: it generates satiety, nausea, discomfort and reward-modulating signals that bias the whole-organism decision without executing it. On the contributory thesis this simply describes how one subject’s affective states are partly generated below the diaphragm. On the partial-subject thesis the same facts describe a negotiation between two loci with partly independent evaluative perspectives, in which the enteric one has no vote on what enters and a considerable vote on what happens next. Eating therefore does not decide between the theses, but it is the clearest place to see what would have to differ for them to come apart.

## Embodiment, temporality, and drive

The gut occupies a privileged position within embodied and enactive theories of mind (Varela et al. 1991, Thompson 2007). Embodied cognition holds that cognition is distributed across brain, body and environment in a tightly coupled dynamical system, and the gut satisfies this description almost paradigmatically. Sensation and action are mechanistically inseparable: peristalsis produces the very signal that regulates peristalsis; motor output is sensory input. Meanwhile the ‘environment’ of the gut is unusually interior. The lumen is topologically outside the organism but physiologically continuous with it, and is populated by a microbial ecology that both shapes and is shaped by enteric activity (Belkaid and Hand 2014, Cryan et al. 2019).

This invites the most serious objection available to a sympathetic reader: that if cognition is distributed across brain, body and environment, there is no motive for singling out one organ system rather than treating the coupled whole as the cognitive agent. The answer is that distribution and multiplication are different claims. Embodied and enactive theories are theories about the vehicle of a single subject’s cognition; they hold that the person thinks with the gut, not that the gut thinks. They are therefore compatible with the contributory thesis and silent on the partial-subject thesis, neither entailing nor excluding it. What would motivate singling out a subsystem is evidence that it satisfies the relevant criteria on its own—that its integration, learning, valuation and closed-loop coupling do not require the remainder of the system, and that much of what it discriminates never becomes available to the remainder at all. Sections 2 and 3 give some reason to think this true of the ENS and of no other organ system; Section 9 asks how the claim could be tested.

Temporality is another criterial property of consciousness on phenomenological accounts (Zahavi 2005). A conscious system has a past and anticipates a future. The gut does both. Cephalic and gastric phases of digestion begin before food arrives, triggered by sight, smell and expectation (Smeets et al. 2010). Chronic dietary history reshapes enzyme expression, mucosal architecture and microbial composition (Cryan et al. 2019). Prolonged abstention from a food class is followed by transient intolerance on re-exposure, a form of forgetting that must be compensated by relearning. These are long-horizon temporal phenomena, not mere reflex.

Drive deserves special attention. Freud’s concept of *Trieb* (Freud 1915)—that which presses and cannot simply be set aside—was named in terms of bodily impulsion, and the German *Trieb*, like the English *drive*, derives etymologically from motor impulsion of the kind exhibited by peristalsis. The etymology is not a pun; it reflects the fact that the primal drives of hunger, satiety, nausea and elimination are organized around enteric sensation and motility. If drive is constitutive of animal consciousness, as Freud, Damasio and Panksepp (Freud 1915, Damasio and Carvalho 2013) have insisted, the gut is its principal organ.

## The immune system as partner in self/non–self-discrimination

A frequent criterion for self-modelling is the ability to distinguish self from non-self. Taken on its own this proves too much. Autopoietic accounts have long held that maintenance of a boundary between self and non-self is constitutive of life as such (Maturana and Varela 1980, Varela et al. 1991), and a bacterium discriminates self from non-self in its restriction–modification and quorum-sensing systems without any temptation to call it conscious. Such discrimination is at most a necessary condition, and what matters is its character: whether it is learned rather than fixed, context-sensitive rather than stereotyped, and integrated with the same network that generates the system’s behaviour. It is on these dimensions that the gut is interesting.

No organ must perform the discrimination more continuously or more subtly. The intestinal epithelium confronts, at every moment, microbial, dietary and endogenous antigens in variable combination. The gut-associated lymphoid tissue (GALT), the largest immune compartment in the body, implements this through Peyer’s patches, mesenteric lymph nodes, secretory IgA, regulatory T cells and dendritic sampling of luminal content (Mowat and Agace 2014, Veiga-Fernandes and Mucida 2016). The ENS is in close cytoarchitectural and signalling contact with GALT; neuroimmune feedback loops are among the most densely studied in contemporary physiology (Belkaid and Hand 2014, Veiga-Fernandes and Mucida 2016). As above, the gut self at issue is minimal: a pre-reflective and pre-linguistic self-differentiation of the kind phenomenologists take to be implicit in any experience whatever, not a represented self-concept of the sort that plausibly requires a cortex (Zahavi 2005, Gallagher and Zahavi 2012).

Were the ENS merely a reflex machine, the sophistication of this immunological dialogue would be wasted. Instead, gut self-modelling appears to be co-computed by neurons, enteroendocrine cells and immune cells, yielding a distributed ‘somatic self’ finer grained than the cephalic self on the chemical axis that matters most to a digestive organ. Food intolerances, food allergies and the irritable bowel syndromes can then be read, in part, as disorders of enteric self/non–self-inference (Drossman 2016, Mayer et al. 2023). Viewed in this light, functional gastrointestinal disorders are less ‘psychosomatic’ than somatocognitive: illnesses of an organ whose inference has gone awry.

## Evolutionary and comparative perspective

Consciousness science increasingly looks to invertebrates for evidence of phenomenal experience beyond the mammalian cortex (Feinberg and Mallatt 2013, Godfrey-Smith 2016, Birch et al. 2020). Three comparative data points are especially relevant.

First, the gut–nervous-system coupling is evolutionarily ancient. Bilaterian animals without a centralized brain, notably lancelets and many early chordates, nonetheless possess elaborate enteric innervation (Holland and Holland 2001), and cnidarians, whose nerve nets lack a central brain altogether, coordinate feeding and digestion through what is in effect a diffuse visceral plexus. Antiquity of the enteric division is not, however, antiquity of consciousness. The prevailing view among those who have worked the question out in detail places the origin of subjective experience in the Cambrian and ties it to the innovations that produced fast directed locomotion, distance senses and centralized brains; Godfrey-Smith (2016), Feinberg and Mallatt (2016), Ginsburg and Jablonka (2019) and Barron and Klein (2016) differ substantially in their criteria but converge on this general location, and no worked-out account places the origin earlier. Two weaker claims survive, and these are the ones we defend: that the machinery of interoceptive regulation is older than centralisation and has retained an unusual degree of autonomy, so that whatever consciousness was later built upon was built on a system already integrating chemical and mechanical information about an interior; and that whichever properties account for consciousness in the Cambrian animals—integration, associative learning, valenced states—the enteric division independently retains a considerable share of them. Neither supports gut-first origins. Indeed the Cambrian account supplies the strongest evolutionary objection to the partial-subject thesis, and we take it up in Section 10.

Second, cephalopods, whose consciousness is now widely accepted (Hochner 2012, Godfrey-Smith 2016, Birch et al. 2020), are in many respects ‘gut-brained’ animals: a substantial fraction of octopus neurons resides in the arms and in a peripherally distributed nervous system whose informational style resembles that of the ENS more than that of the mammalian cortex (Fiorito and Scotto 1992, Hochner 2012). Jellyfish and other cnidarians exhibit sleep-like states, learning and stimulus-specific responses using nerve nets topologically analogous to enteric plexuses (Nath et al. 2017). Godfrey-Smith (2021) draws from the octopus case a careful moral: integration may be a matter of degree, so that the alternatives are not simply one subject or nine. That is the moral we borrow, rather than the claim that the octopus is a settled precedent for anything in the gut.

Third, within mammals, the ENS derives ontogenetically from vagal and sacral neural crest cells that migrate and organize largely independently of the CNS (Bouso et al. 2018). That this system is phylogenetically first and ontogenetically self-assembling is consistent with its being a locus of primary rather than derived experience: the scaffold on which cephalic consciousness was later built.

## Clinical and pharmacological signatures

Several clinical observations are readily accommodated by a consciousness-like account of the gut and awkward under a reflex-only account.

Classical serotonergic psychedelics reliably produce nausea and visceral discomfort among their most frequent acute effects (Nichols 2016, Carhart-Harris and Goodwin 2017), acting on 5-HT2A and related receptors that are heavily expressed in the ENS and not only in the cortex. Interpreted as a reorganisation of interoceptive inference, psychedelic nausea may be less non-specific than it appears: a perturbation of enteric predictive control that parallels, in a far simpler system, the perturbation of cortical self-modelling these drugs produce above the neck. This reading is fully compatible with the contributory thesis and in fact more naturally stated within it. What would speak for the partial-subject thesis is a dissociation—between the valence of the enteric response and that of the concurrent central experience, or persistence of the enteric signature under conditions in which the central one is abolished. The route of administration is suggestive here: oral psilocybin and ayahuasca engage enteric 5-HT3 signalling on the way to the brain, producing nausea or frank emesis in a substantial fraction of sessions, whereas intravenous administration bypasses the gut and the nausea largely disappears (Carhart-Harris et al. 2012). The visceral dimension of the experience thus tracks gut exposure rather than total drug level, a pattern hard to reconcile with the gut as a mere conduit.

Glucagon-like peptide-1 (GLP-1) receptor agonists (Drucker 2018) provide a second convergent line. Their efficacy in suppressing food intake does not reduce to peripheral hormone activity; it depends on gut–brain vagal signalling and on reshaping anticipatory and reward predictions that implicate both enteric and cephalic representations, and GLP-1 agonism modifies mood, anxiety and compulsivity alongside appetite (Drucker 2018). What is transmitted is therefore richer than a scalar report of nutrient load. This bears on how much the gut contributes to affect; it does not by itself bear on whether the gut has affect.

Chronic functional GI disorders—irritable bowel syndrome, functional dyspepsia, cyclic vomiting—are defined, in the Rome IV criteria (Lacy et al. 2016), by patterns of symptom generation that do not reduce to discrete anatomical lesions. They are better described as disorders of the gut’s interoceptive inference, and they respond disproportionately to interventions that modulate enteric self-modelling: cognitive behavioural therapy, gut-directed hypnotherapy and central neuromodulators (Lacy et al. 2016, Mayer et al. 2023). We put this no more strongly than the data allow: what the clinical picture supports is that enteric regulation is model-based and can be miscalibrated, which is a claim about inference rather than about experience. Whether a miscalibrated model is a suffering one is exactly the question at issue, and Section 9 rather than Section 8 is where it has to be settled.

## Telling the two theses apart

The two theses were distinguished conceptually in Section 1.1. The harder question, and the one we take to be the principal methodological contribution of this paper, is how they might be told apart by observation. We set out five lines of investigation. None is decisive alone; together they constitute a research programme, and each has the property that the two theses make different predictions about its outcome.

**(i) Informational segregation.** On the contributory thesis, what matters about enteric processing is what eventually reaches the central nervous system by vagal, spinal or humoral routes; enteric activity that never leaves the abdomen is no better a candidate for experience than the activity of a kidney. It is therefore significant that the greater part of enteric afferent traffic terminates locally in the myenteric and submucosal plexuses and never reaches the brain (Furness 2012, Spencer and Hu 2020). The measurement to make is to characterize the discriminations the ENS demonstrably performs—among amino acid species, bile acid profiles, bacterial metabolites, osmolarities, and wall tensions at different loci—and to determine what fraction of them is recoverable from vagal and spinal afferent traffic. Discriminations the gut makes that are not represented centrally cannot be contents of the whole-organism subject, since that subject has no access to them by any route. This does not show that they are contents of anything. It does show that the contributory thesis leaves them unaccounted for, and it identifies the population of states about which the partial-subject question is actually being asked.

**(ii) Persistence under disconnection.** The partial-subject thesis has a consequence that should be stated plainly rather than hedged: it implies that a surviving, perfused, extrinsically denervated but internally intact enteric preparation would retain whatever minimal phenomenality it has, much as an isolated perfused brain would. This is uncomfortable, and it is also the thesis’s most useful feature, because it is testable in the coin of markers rather than of reports. The relevant preparations already exist: chronically isolated intestinal loops, extrinsically denervated segments, *ex vivo* perfused colon and, in the clinic, high spinal cord injury and truncal vagotomy (Furness et al. 2014, Spencer and Hu 2020). The question to put to them is whether the marker cluster on which the candidacy claim rests—nociceptive sensitisation, conditioning and habituation, opioid-modulated antinociception, context-dependent gain, and integration of chemosensory with mechanosensory information into flexible output (Birch 2024)—survives the loss of central input, degrades gracefully, or collapses. Collapse would be strong evidence for the contributory thesis, since it would show that the apparent sophistication of enteric processing is a property of the loop through the brain. Survival would not establish a second subject, but it would remove the most economical alternative explanation.

**(iii) Dissociation and conflict.** Conflict by itself proves nothing. A single subject routinely harbours incompatible drives and desires—intrapsychic conflict is the normal condition of a unified subject rather than a sign of division (Freud 1915)—and no one takes hunger competing with restraint to indicate two experiencers. What distinguishes the unified case is that the competing states are compared, weighted and resolved within one evaluative space; two loci can stand in a conflict that is not resolvable in this way, because neither has access to the other’s evaluation. Enteric–central conflicts of roughly the right shape are common in the clinic: the food that is wanted and reliably rejected; the conditioned visceral aversion that survives full propositional knowledge of its irrationality; the irritable bowel in which the patient’s assessment of a meal and the gut’s are stably opposed. The methodological template for taking these more seriously than the customary contrast between cognition and physiology is the split-brain literature, in which behavioural dissociation was argued to indicate more than one stream of experience within a single skull (Bayne 2010, Schechter 2018). That literature also supplies the warning: dissociation of processing is not automatically multiplication of subjects, and after 50 years its interpretation remains contested. What would carry weight is not the existence of a conflict but its structure—evidence that the enteric evaluation is integrated across enteric contents while remaining unintegrated with the central one. That is what a second locus predicts and what a merely encapsulated input channel does not.

**(iv) Where the maxima of integration lie.** As set out in Section 4.1, IIT alone among the frameworks addresses the partial-subject question directly, since whether the ENS is a separate complex or part of a larger one simply is the question of whether there is a second subject (Oizumi et al. 2014). Approximate integration measures can be computed from multi-electrode and optical recordings of enteric networks and, more importantly, from simultaneously recorded enteric and central activity, in order to ask whether the joint system or the enteric system alone constitutes the local maximum. The available measures are crude and their relation to Φ proper is disputed, but the question is well posed and the recordings are becoming feasible.

**(v) Pharmacological double dissociation.** Differences in route of administration and in receptor distribution allow the enteric and central arms of a drug’s action to be separated. Peripherally restricted agonists and antagonists, comparisons of oral with parenteral administration, and comparisons of intact with vagotomized preparations can be used to ask whether the enteric markers of interest track enteric drug exposure or total exposure. The psychedelic and GLP-1 observations of Section 8 are suggestive here but were not designed for the purpose. A study designed for it would hold central exposure constant while varying enteric exposure, and would measure the enteric marker cluster rather than the subjective report.

We end with a limit rather than a promise. It is possible that no observation settles the partial-subject question, and that what remains after all five lines of work is the problem of other minds in an unusually sharp form: a system with properties we take to be sufficient elsewhere, which by construction can tell us nothing. If that is where the question ends, it will not be for want of having been asked precisely.

## Objections

Four objections deserve explicit address.

**First, the absence of report.** The gut cannot narrate its experience. The limit is genuine, but it is shared with every non-linguistic creature to which the field routinely attributes consciousness, from octopuses to human neonates; absence of report is not absence of phenomenology (Godfrey-Smith 2016, Birch et al. 2020). Nor is language what grounds our recognition of other minds. That ground, in the phenomenological tradition, is empathy—Husserl’s and Stein’s *Einfühlung*—the pre-linguistic apprehension of another’s bodily expressivity as the expressivity of a subject (Zahavi 2005, Zahavi 2014). Scheler put the point most strongly: the other’s subjectivity is not inferred behind their comportment but perceived in and through it, which is why we do not ordinarily experience other minds as hidden. Language refines this attunement; it does not constitute it.

The objection therefore turns on a narrow construal of *report*. If a report must be a linguistic act, the gut makes none. If a report is any expression that is expressive of the state it reports, the gut is among the more eloquent organs in the body. Nausea, urgency, cramping, satiety, the aversion that follows a poisoning and the ease that follows a needed meal are not inferred by the person from instrument readings; they are undergone, and they arrive with valence, insistence and a rough location. On this broader construal the gut expresses continuously, and it expresses to the very subject with whom the partial-subject thesis says it shares a body.

Two cautions keep this from proving too much. Expression in the broad sense is cheap: a thermostat’s click expresses its state, and so does a wilting leaf; what matters is not that a state is legible from outside but whether it is undergone from inside, and no amount of expressive richness settles that. And the phenomena just listed are, on the contributory thesis, straightforwardly the person’s own experiences of their own body, the ease of reading them being fully explained by their being ours. The question that connects this objection to Section 9 is therefore not whether the gut expresses, but whether anything it discriminates fails to become the person’s own state while remaining organized, valenced, and integrated on the enteric side. What the objection does establish is that the ordinary route by which we confirm consciousness in others—empathic apprehension, corroborated by convergent behaviour and eventually by testimony—is closed here at its last step, so that the weight of the argument falls on the indirect markers of Section 4 and the tests of Section 9.

**Second, the absence of spatial locomotion.** Gut consciousness, if it exists, is constrained to act within its own lumen; it cannot move through external space, orient toward distal targets or construct allocentric maps. This circumscribes the form of consciousness involved, which lies closer to a diffuse visceral awareness than to the agentic cognition of primates, but does not disqualify it: not all conscious systems must be ambulatory. It does place the hypothesis in tension with the Cambrian accounts of Section 7, on which experience arose in the service of action in external space—which is why we regard the evolutionary literature as constraining our proposal more than supporting it.

**Third, the suspicion of anthropomorphic projection** inflected by cultural factors: disgust, social subordination and the traditional opposition of ‘higher’ brain to ‘lower’ gut. We suggest the reverse: the long neglect of gut consciousness reflects such biases, while the neurobiological and information-theoretic facts point the other way. An impartial application of IIT at least makes the gut a candidate to be assessed rather than one to be dismissed on anatomical grounds. Panpsychism provides no support here, since a view on which everything is conscious privileges the gut no more than the pancreas (Goff 2019).

**Fourth, the unity of consciousness.** The most substantial objection is not that the gut is too simple but that the proposal violates a deeply held and well-motivated assumption: that a person has one phenomenal field, not two. It helps to distinguish what may be meant by unity. *Phenomenal unity*, strictly, is subsumption: at a given moment a subject’s experiences are had together, as parts of one total experiential state (Bayne 2010). *Unity of subject* is the further claim that there is exactly one bearer of experience per organism. *Access unity* is the availability of contents to a single system of report and deliberate control. The three come apart. Access unity plainly fails for the gut, since most enteric discrimination never reaches report, and no one takes this to threaten anything. Unity of subject is what the partial-subject thesis denies. Phenomenal unity is the interesting case, and here a third possibility deserves mention.

Suppose there are two loci. It does not follow that there are two sealed fields. The coupling between them is dense, continuous and bidirectional, and on the contributory evidence constitutive rather than incidental: much of what appears in the cephalic field is enteric in origin, and much of what the gut does is set by descending and humoral signals carrying the organism’s situation. A field so constituted might be neither one subject’s nor two subjects’ in the ordinary sense, but jointly constituted between them—the phenomenal analogue of what phenomenology calls intersubjective constitution, in which a shared world is neither yours nor mine but is achieved between us (Zahavi 2014). We raise this as a possibility worth taking seriously rather than a position we can currently defend. It matters because it changes the empirical question from ‘one or two?’ to how much of the joint field each locus constitutes and how much either retains when the coupling is cut, which is what the tests of Section 9(ii) and 9(iv) measure. It also inherits, in reverse, the combination problem familiar from panpsychism (Chalmers 2017): a proposal of this kind owes an account of where one locus ends and the other begins, and of why their coupling integrates their contents to the degree it does rather than more or less. These are difficult questions rather than conceptual impossibilities.

Two further responses. The disturbance many readers feel is not itself evidence, and the history of consciousness science contains results—split-brain dissociation above all—that were unsettling and turned out to be worth taking seriously (Bayne 2010, Schechter 2018). And the objection has no force at all against the contributory thesis, which requires no departure from unity and which carries most of the practical consequences we draw. A reader who accepts our neurobiology and rejects our metaphysics is welcome to the former; we have tried to keep the two separable.

## Conclusion and future directions

We have argued that contemporary theories of consciousness identify the ENS as a candidate substrate for an ‘animal’ mode of consciousness dominated by interoceptive feel, drive and affect. The convergence is partial rather than uniform: integrated information theory, predictive processing and enactive accounts each make the gut a candidate on their own criteria, whereas global workspace theory, applied strictly, does not currently support the claim, and Nagel’s criterion is not a further vote but a statement of what the others are theories of.

The hypothesis is testable. Φ-style measures could be approximated from high-resolution enteric recordings; global-workspace signatures could be sought in long-range coordinated enteric activity; interoceptive prediction error could be tracked in vagal afferents during meal ingestion and pharmacological manipulation. Clinically, reconceptualising functional GI disorders as disorders of gut self-modelling suggests new intervention targets at the enteric–immune interface. Theoretically, gut consciousness invites a re-examination of the evolutionary origins of experience and of the relationship between neural and non-neural contributions to mind, including the microbiome whose metabolic voice participates in the very signals the gut integrates. Above all, the programme requires that the two theses of Section 1.1 be kept apart. The contributory thesis—that a large part of what it is like to be an animal is made below the neck—we take to be well supported and consequential in its own right. The partial-subject thesis—that there is a second and far simpler locus of experience in the abdomen—remains open, and we have tried to say what it would take to close it in either direction.

What does it feel like to be a gut? We cannot know from outside. But to ask the question seriously, and to study the ENS as the candidate organ of an overlooked mode of consciousness, is a scientifically productive move. The head has been the protagonist of consciousness research for good reason, but it need not remain the only one.
